# Intragenomic and Interspecific Polymorphism of 35S rDNA Internal Transcribed Spacer 1 (ITS1) in *Selenicereus* and Related Species (Cactaceae: Cactoideae: Phyllocacteae)

**DOI:** 10.3390/ijms27167351

**Published:** 2026-08-17

**Authors:** Alexander V. Rodionov, Yuri G. Kalugin, Evgenia L. Romanova, Peter M. Zhurbenko, Elizaveta O. Punina, Alexander G. Dyomin, Ivan N. Shulzhenko, Alexander A. Gnutikov, Nikolai N. Nosov

**Affiliations:** 1 Komarov Botanical Institute of the Russian Academy of Sciences, St. Petersburg 197376, Russia; avrodionov@binran.ru (A.V.R.); eromanova@binran.ru (E.L.R.); petr.zhurbenko@weizmann.ac.il (P.M.Z.); epunina@binran.ru (E.O.P.); adyomin@binran.ru (A.G.D.); ishulzhenko@binran.ru (I.N.S.); a.gnutikov@vir.nw.ru (A.A.G.); nnosov@binran.ru (N.N.N.); 2 Faculty of Biology, St. Petersburg State University, St. Petersburg 199034, Russia; 3 Weizmann Institute of Science, Rehovot 7610001, Israel; 4 Biological Station Rybachy, Zoological Institute, Russian Academy of Sciences, Rybachy 238535, Kaliningrad Oblast, Russia; 5 N.I. Vavilov All-Russian Institute of Plant Genetic Resources (VIR), St. Petersburg 190000, Russia

**Keywords:** 35S rDNA, Cactaceae, Caryophyllales, 5.8S rRNA pseudogenes, intragenomic rDNA polymorphism, DNA barcoding, molecular phylogeny

## Abstract

Locus-specific Illumina NGS was used to analyze the polymorphism of the partial 18S rDNA, spacer ITS1, and partial 5.8S rDNA sequences across 25 cactus species (53 accessions) of the subtribes Hylocereinae and Echinocereinae. To ensure data reliability, only ribotypes (zero-radius operational taxonomic units, ZOTUs) with a minimum read depth of 10 reads within each genome-read pool were retained. A single ribotype was identified in 18 analyzed genomes. In 23 accessions, two or three ribotypes were detected, typically consisting of a major ribotype (98–99%) and one or two minor ribotypes. In the remaining samples, two distinct ribotypes were present in approximately equal proportions, suggesting that these individuals are recent hybrids. Ribotype patterns clearly distinguish the genus *Deamia* from *Selenicereus*, *Hylocereus*, and *Epiphyllum*. Putative pseudogenes with deletions in the 18S and 5.8S rDNA, as well as deletions and insertions in the ITS1 spacers, were detected. Their abundance was generally low (usually <1%), except in two *Deamia* species (5–15%). In ITS1 of some minor ribotypes, deletions eliminated cleavage sites B1, B2, and A3, which are important for pre-rRNA processing. Consequently, the products of these putative pseudogenes could not participate in ribosome formation. In addition, pseudogenic copies exhibited a strong transition bias and A/T enrichment in the ITS1 region.

## 1. Introduction

Genes encoding the RNA components of ribosomes—ribosomal RNAs (rRNAs)—are indispensable structural and functional elements of both eukaryotic and prokaryotic genomes [1,2,3]. In animals, these clusters are designated as 45S and 5S rRNA genes, whereas in plants and yeasts, they are represented by 35S and 5S rRNA genes [4]. The internal transcribed spacers, ITS1 and ITS2, constitute the most variable regions of the 45S/35S rRNA gene cluster. Consequently, they serve as the most widely used molecular markers in plant and fungal biology for DNA barcoding and phylogenetic reconstructions at the level of genera, sections, and tribes [4,5,6,7].

In plant genomes, 35S rRNA genes are organized as tandem repeats, with copy numbers ranging from several hundred to several thousand per haploid genome [8,9,10,11]. These repeats are localized within specialized chromosomal regions known as nucleolus organizer regions (NORs). Plant genomes may contain one or multiple NORs; the median number of NORs per haploid genome is two in land plants [12].

Intragenomic polymorphism within the 35S rDNA cluster can arise from several independent evolutionary events. A major source of polymorphism is interspecific hybridization [13,14,15,16]. Notably, even in ancient allopolyploids, ancestral rDNA lineages derived from distinct parental species can coexist and persist for millions of years [14]. A second driving factor is spontaneous mutagenesis, whereby individual gene copies accumulate single-nucleotide polymorphisms (SNPs) and insertions/deletions (indels) due to replication errors or inaccurate repair of double-strand breaks [17,18]. A third potential cause, whose molecular basis remains poorly understood, is the horizontal transfer of rDNA [19,20] and the insertion of foreign sequences into the NORs [21,22].

Despite these diversifying factors, 35S rDNA arrays, like other tandemly repeated sequences in eukaryotic genomes, typically evolve in a concerted manner [14,23]. As a result, the observed level of intragenomic 35S rDNA sequence polymorphism is generally lower than would be expected if each gene copy accumulated mutations independently. This pattern suggests that 35S rDNA arrays may evolve according to a “birth-and-death” model [14,24]. Although the precise genetic mechanisms underlying this model remain to be fully elucidated [14], they are likely associated with the deletion of pre-existing sequences followed by the compensatory replication of specific rDNA copies [25,26,27].

It Is probable that the prevailing consensus regarding the high degree of homogeneity among eukaryotic rRNA genes has been overestimated. For instance, approximately 50% of the 35S rRNA gene copies across six *Cycas* L. species have been identified as pseudogenes [28,29]. Similarly, about 60% of the sequenced ITS arrays in the genus *Lycium* L. (Solanaceae Juss.) have been interpreted as pseudogenic spacers [30]. Furthermore, an investigation of ITS1 and ITS2 polymorphism in 90 species of the genus *Asclepias* L. (Apocynaceae Juss.) revealed that 61% of sites in ITS1 and 57% of sites in ITS2 exhibited high polymorphism, with mutations occurring in at least 10% of the reads for these species [31].

Several studies have documented elevated variability within the 35S rRNA gene cluster in Cactaceae Juss. Pseudogenes are exceptionally abundant within the NORs of *Mammillaria* Haw., where they comprise approximately 90% of the total gene pool in these regions [32,33]. Pseudogenic copies characterized by extensive deletions in ITS2 and elevated substitution rates in 5.8S rDNA have also been described in several cactus species belonging to the genera *Lophocereus* (A. Berger) Britton & Rose and *Pachycereus* (A. Berger) Britton & Rose (Pachycereeae) [34]. While pseudogenes were inferred to be numerous in the genomes of *Tephrocactus molinensis* (Speg.) Backeb. And *Pereskiopsis aquosa* (F.A.C. Weber) Britton & Rose, they appeared to be absent from the NORs of other Opuntioideae Burnett representatives [35,36]. Conversely, all ITS1–5.8S rDNA–ITS2 arrays cloned and sequenced from the genus *Selenicereus* (A. Berger) Britton & Rose (Hylocereeae Buxb.) by Plume et al. [37] exhibited high polymorphism and carried mutations within two or three highly conserved regions of the 5.8S rDNA sequence, suggesting they likely represent pseudogenes [38].

The aim of the present study was to assess the extent of Intragenomic rDNA polymorphism and, specifically, to quantify the prevalence of pseudogenes within the 35S rDNA arrays of *Selenicereus* and related species. To achieve this, we applied locus-specific Next-Generation Sequencing (NGS) on the Illumina platform to sequence the 18S rDNA (fragment)–ITS1–5.8S rDNA (fragment) region across 25 cactus species of the tribe Phyllocacteae. We demonstrate that within the genomes of the examined *Selenicereus*, *Epiphyllum* Haw., *Hylocereus* (A. Berger) Britton & Rose and *Deamia* Britton & Rose species, the read pool (only ZOTUs represented by at least 10 reads per genome) includes ribotypes carrying deletions in the coding 18S and 5.8S rRNA regions, alongside extensive insertions and deletions (indels) within ITS1. However, such variants generally accounted for less than 1% of total reads. A notably higher proportion of pseudogenes, ranging from 5% to 15%, was observed exclusively in the genomes of two *Deamia* species. The identified pseudogenes carried deletions and multiple mutations in the 18S and 5.8S rDNA, as well as insertions and deletions in ITS1, affecting sites A2, A3, and B1, which are necessary for the correct processing of pre-rRNA. We propose that disruption of pre-rRNA processing of mutant 35S rRNA genes contributes to genomic homeostasis by reducing the likelihood of aberrant ribosome formation.

## 2. Results

We sequenced the 18S rDNA (partial)–ITS1–5.8S rDNA (partial) region, extending from the AACCTTAT motif in 18S rDNA to the AACGCAGC motif in 5.8S rDNA, in 25 cactus species of the tribe Phyllocacteae (see the full list in the Section 4 and in Table 1).

**Figure 1:**
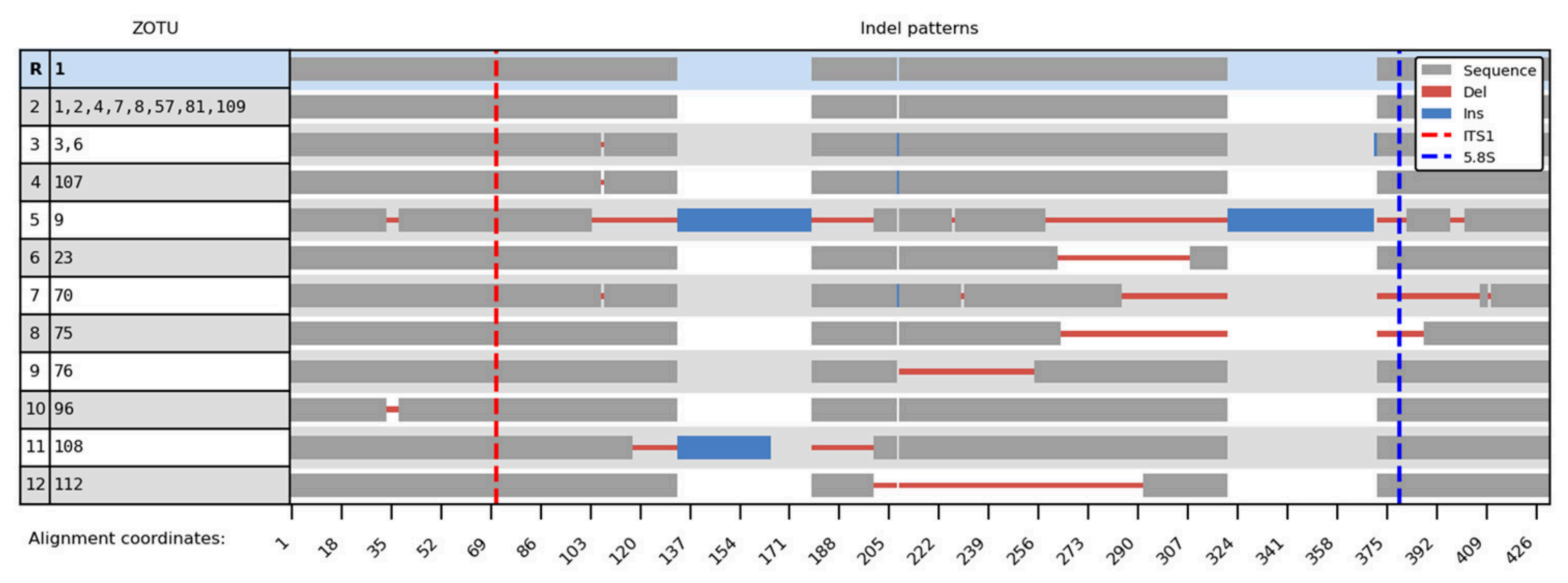
Insertions and deletions in the sequenced 18S rDNA–ITS1–5.8S rDNA region of 25 species and subspecies of the tribe Phyllocacteae. Red and blue dashed lines indicate the boundaries between 18S rDNA and ITS1 and between ITS1 and 5.8S rDNA, respectively.

To ensure robustness, only ZOTUs represented by at least 10 reads in the ITS1 dataset of each genome were retained for further analyses. In total, 19 such ZOTUs were identified across all examined genomes (Table 1). The NGS sequence data for the ZOTUs have been deposited in NCBI GenBank (see: Section 4). The sequence lengths of these ZOTUs ranged from 241 bp to 333 bp, with a modal length of 332 bp. This variation resulted primarily from large deletions and insertion events. Following multiple sequence alignment, the total alignment length was 438 bp (Figure 1).

**Figure 2:**
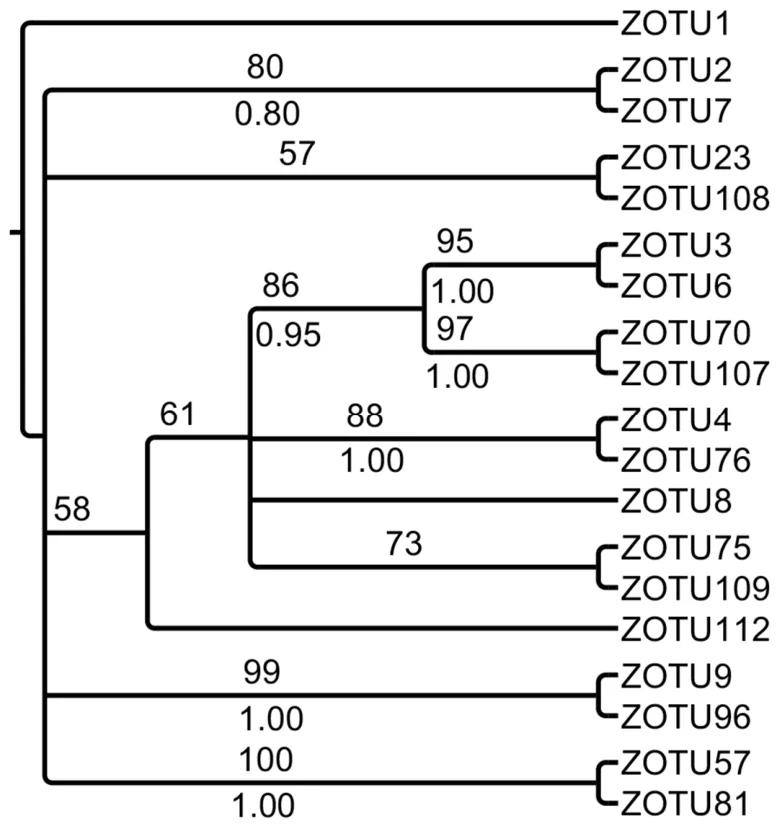
Evolutionary relationships among 19 ITS1 ribotypes (ZOTUs). The number above the branch is the bootstrap index (maximum likelihood method); the number below indicates posterior probability in Bayesian inference. The ribotypes were identified in rDNA reads from 25 species and subspecies of the tribe Phyllocacteae; each ribotype was represented by at least 10 reads. ZOTU1 was used as the root of the tree.

A phylogenetic tree illustrating the sequence similarity and potential evolutionary relationships among the investigated variants is presented in Figure 2.

With one exception, major variants (defined as ZOTUs representing >5% of reads) were highly conserved and differed from ZOTU1 by only 1 to 8 SNPs. The remaining ZOTUs represented derivatives of the major ribotypes carrying large deletions or insertions (Figure 1, Table 2). Notably, this included the major ribotype of *Deamia* (ZOTU9), which exhibited two extensive insertions and six deletions (Figure 1). These two insertions showed homology to unannotated regions of the mitochondrial genomes of *Selenicereus monacanthus* (hort. Ex Lem.) D.R. Hunt (NC_081468), *Lophophora williamsii* (Lem. Ex J.F. Cels) J.M. Coult. (PZ264132.1), and *Mammillaria huitzilopochtli* D.R. Hunt (NC_080974). This ribotype was relatively abundant in four samples representing two *Deamia* species, where it accounted for 5% to 15% of the total reads. In contrast, this ribotype was not detected in the genomes of *Selenicereus* and *Epiphyllum*. Another distinct variant, ZOTU108, was characterized by a 32-nucleotide insertion (Figure 1). This variant represented a minor ribotype in *Selenicereus pteranthus* f. *macdonaldiae* (Hook.) Ralf Bauer S44. This ribotype arose from a tandem insertion into the ancestral ITS1 region of two translocated duplications derived from the 5′ end of the 18S rDNA (corresponding to positions 58–69 and 27–46 in our alignment). These insertions likely originated through stochastic mutational processes followed by the subsequent intragenomic amplification of the mutated rRNA cistron variant.

Table 2 presents the summary of the rDNA polymorphism analysis across the 53 examined cactus samples, indicating the numbers of single-nucleotide polymorphisms (SNPs, including transitions and transversions), deletions, and insertions that differentiate each ZOTU from ZOTU1. Because every nucleotide can undergo one type of transition (e.g., G → A) and two types of transversion (G → C or G → T), the aggregate transition-to-transversion rate ratio (ti/tv) has a null expectation of R = P:Q = 0.5 [39]. In our dataset, the ti/tv ration exceeded the null expectation by approximately fivefold in 18S rDNA and sixfold in ITS1 regions (Table 2). By contrast, the short 5.8S rDNA fragment exhibited a ratio well below the null expectation (R = 4:18 = 0.2). Notably, as a result of mutations within the ITS1 region of the putative pseudogenes (Table 2), an A:T base pair was replaced by a G:C base pair in only 21 instances, whereas a G:C pair was substituted by an A:T pair in 71 instances. Thus, a clear trend toward A/T enrichment was observed in the ITS1 spacer of 35S rRNA pseudogenes compared to their putatively functional 35S rDNA copies. The average GC content of ZOTU1–ZOTU8, which likely represent functional 35S rRNA genes, was 63.2%, whereas the remaining ZOTUs exhibited an average GC content of 58.9%. Consequently, putative pseudogenes exhibited a GC content approximately 4 percentage points lower than that of putatively functional ribotypes.

Among the putative pseudogenes (Figure 1, Table 2), several sequences carried extensive deletions within the ITS1 region. Specifically, the ribotypes ZOTU9, ZOTU70, and ZOTU75 lacked the B1 processing site (cac/GACTCTCG) located at the ITS1–5.8S rDNA boundary. This site is required for the precise cleavage of 27S-A3 pre-rRNA and the subsequent formation of mature 5.8S rRNA [3,40]. Furthermore, ZOTU23, ZOTU76, ZOTU108, and ZOTU112 also harbor large deletions within ITS1 (Table 2, Figure 1) that encompass the A2 (a/aagacccgcgaa) and/or A3 (aagg/aaca) processing sites, both of which are essential for 40S pre-rRNA processing [3,40]. In addition to these structurally disrupted variants, we infer that ZOTU51 and ZOTU57 also represent pseudogenes owing to the unusually high number of SNPs present in their 5.8S rDNA sequences.

**Figure 3:**
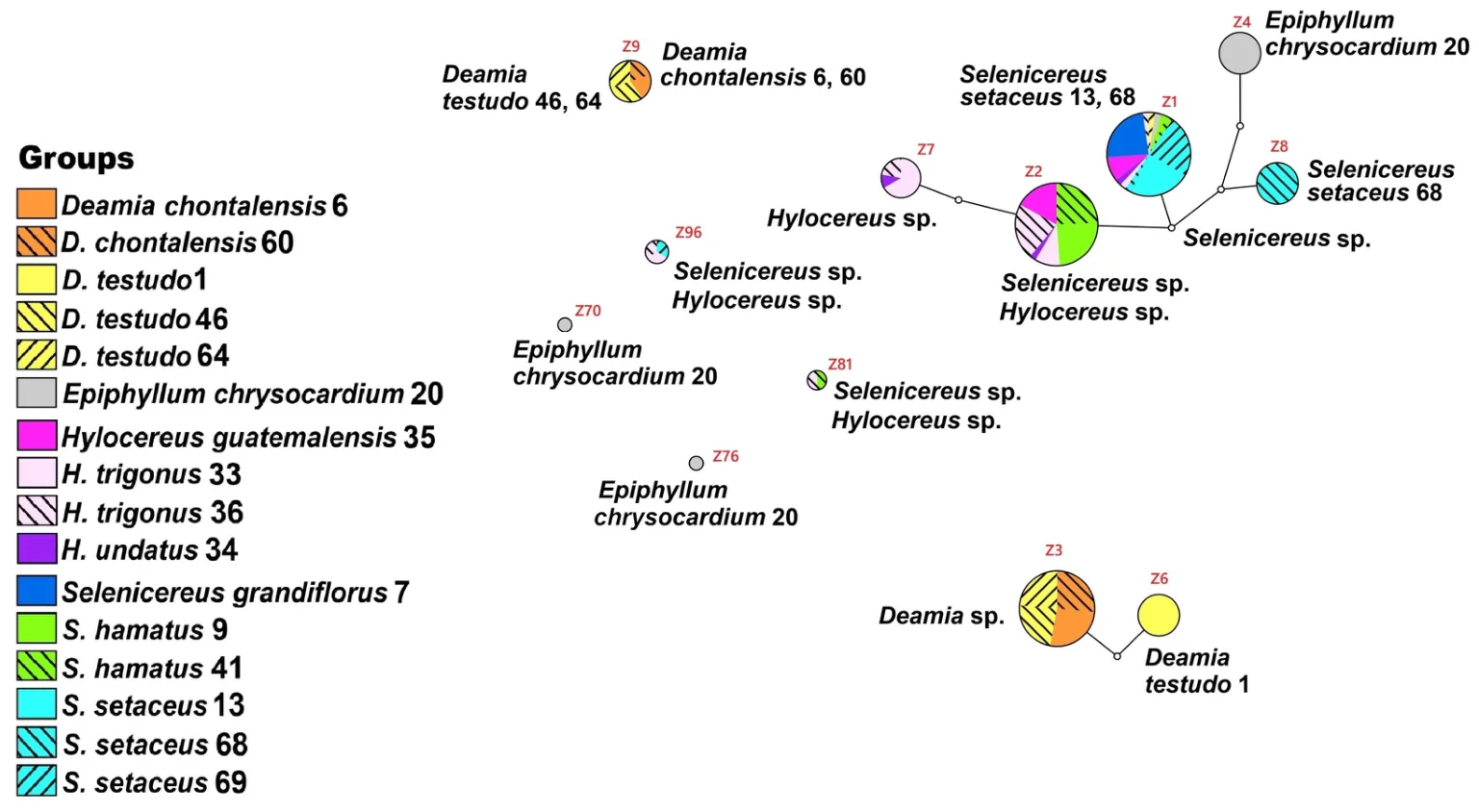
Network of ITS1 ribotypes detected in the rDNA of *Deamia*, *Hylocereus*, *Epiphyllum*, and selected *Selenicereus* species. The network was constructed using TCS v1.21 based on the statistical parsimony algorithm [41] and visualized with tcsBU [42]. Circle radii and sector sizes are proportional to the relative abundance of each ribotype within a given genome (relative units: 0.1–9.9% = 1; 10.0–19.9% = 2; 20.0–39.9% = 3; 40.0–59.9 = 4; 60.0–79.9 = 5; 80.0–99.9% = 6).

**Figure 4:**
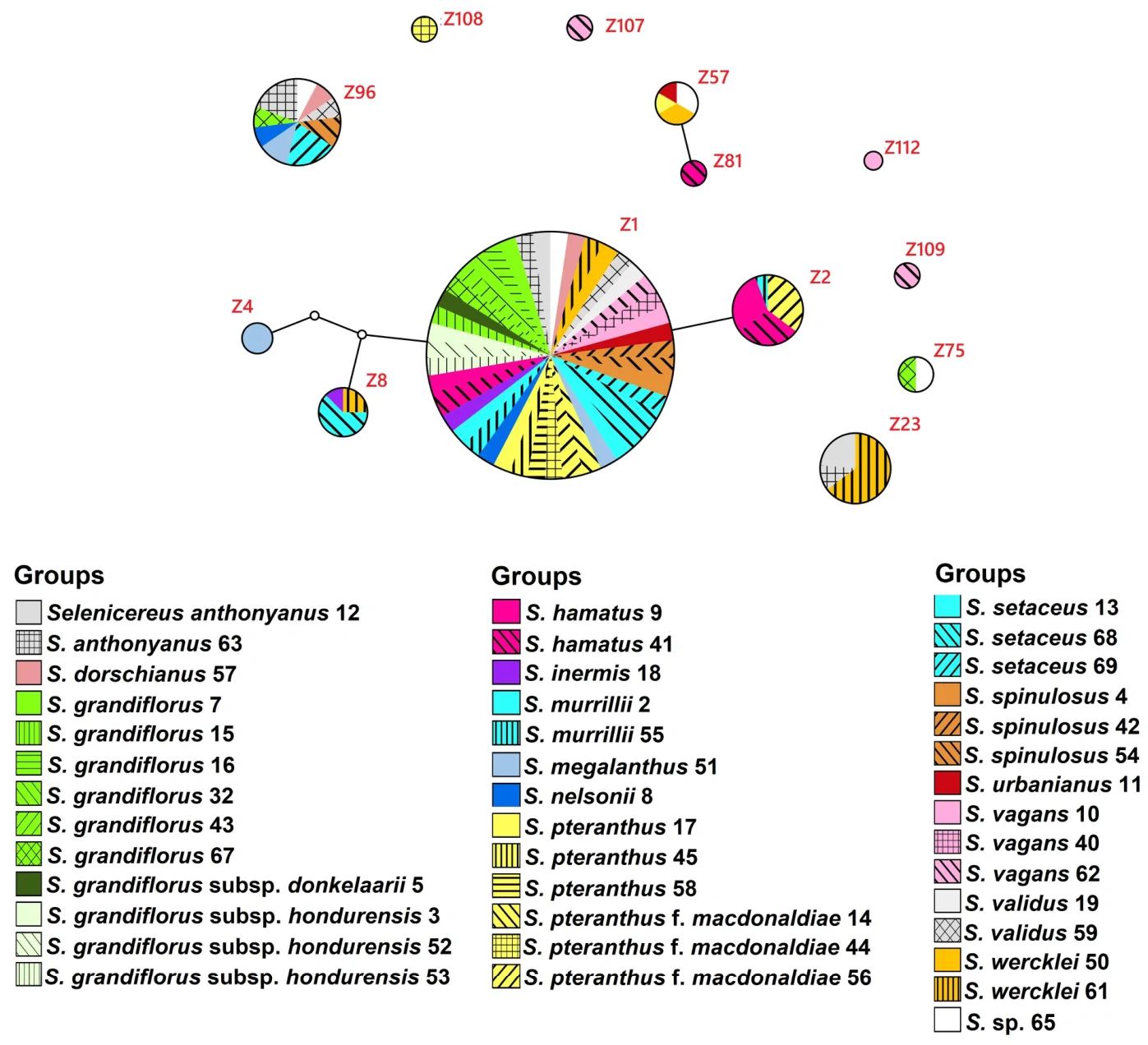
Network of ITS1 ribotypes detected in the rDNA of *Selenicereus* species. See also Figure 3. Circle radii and sector sizes are proportional to the relative abundance of each ribotype within a given genome (relative units: 0.1–9.9% = 1; 10.0–19.9% = 2; 20.0–39.9% = 3; 40.0–59.9 = 4; 60.0–79.9 = 5; 80.0–99.9% = 6).

**Figure 5:**
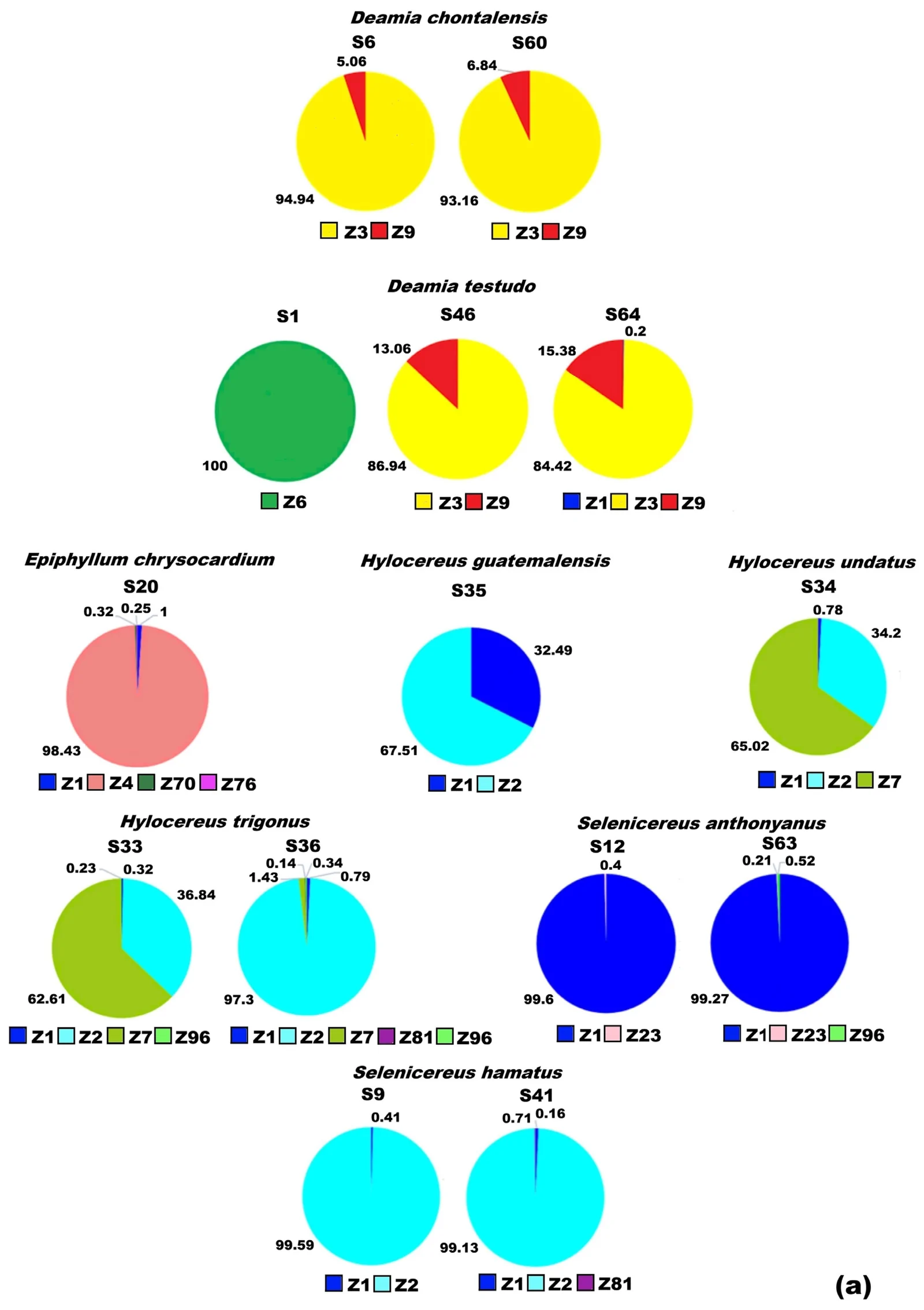

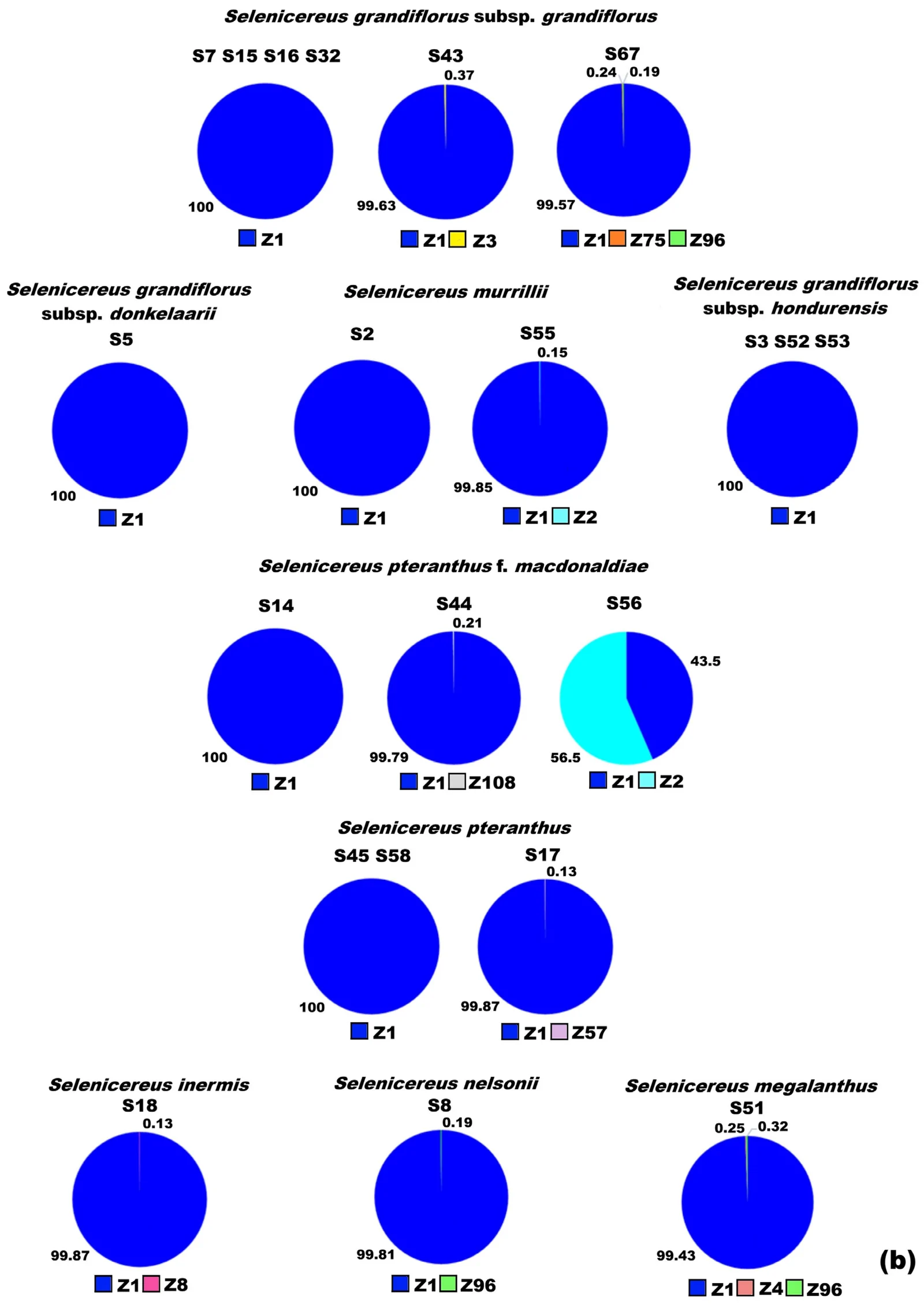

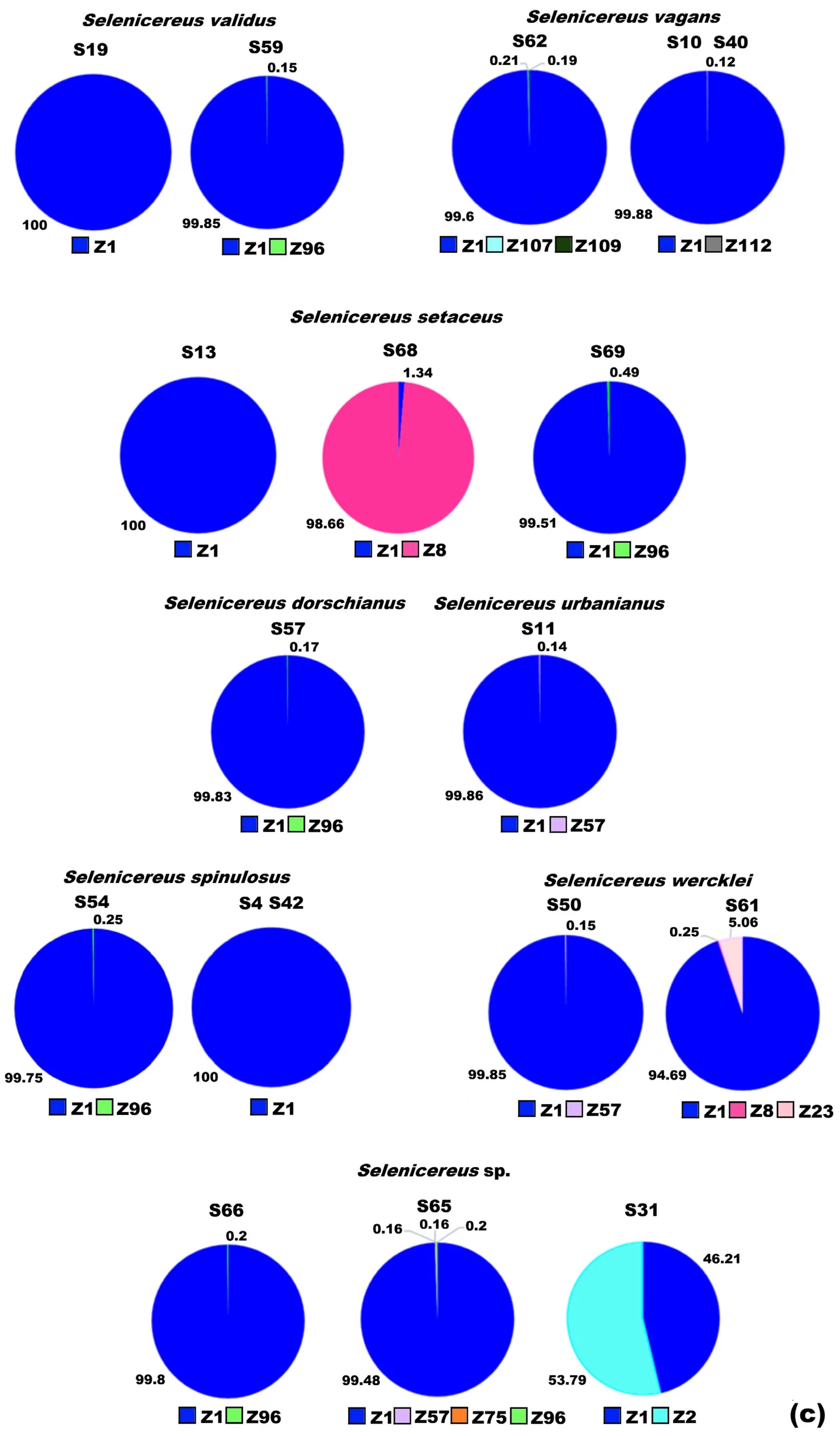
(**a**–**c**). Intragenomic ribotype polymorphism in species of the tribe Phyllocacteae. Sector sizes are proportional to the percentage of reads represented by each ribotype within a given genome.

Intragenomic ribotype diversity was generally low among the analyzed samples (Table 1). A single, uniform ribotype was identified in 18 of the examined samples. In 23 species, two or three ribotypes coexisted, typically consisting of a major ribotype accounting for 98–99% of reads and one or two minor variants (Table 1, Figure 3, Figure 4 and Figure 5). In the remaining cases, two distinct ribotypes were detected in approximately equal proportions. For instance, in sample S56 (accessioned in the collection as *Selenicereus pteranthus* f. *macdonaldiae*), 46.5% of the reads corresponded to ZOTU1—the dominant ribotype of *Selenicereus* spp.—whereas 53.5% of the reads belonged to ZOTU2, which is the major ribotype of *Selenicereus hamatus* (Scheidw.) Britton & Rose and *Hylocereus* spp. (Figure 3). This nearly equal representation of two major ribotypes strongly suggests a hybrid origin, likely representing a first-generation hybrid.

Comparative analysis of the ribotype patterns across different species and genera (Figure 3, Figure 4 and Figure 5) demonstrated that ZOTU1 was the dominant ribotype in the majority of *Selenicereus* species. Only two exceptions were observed: in both examined samples of *Selenicereus hamatus*, the major ribotype was ZOTU2 rather than ZOTU1, while *Selenicereus setaceus* (Salm-Dyck) A. Berger ex Werderm. S68 was dominated by ZOTU8. ZOTU2 differed from ZOTU1 by a single transition, whereas ZOTU8 differed by one transition and one transversion within ITS1. A distinct ribotype profile was identified in *Epiphyllum chrysocardium* Alexander, where the predominant variant was ZOTU4 (differing from ZOTU1 by two transitions and one transversion), accompanied by ZOTU1 as a minor component (1% of reads) and the pseudogenes ZOTU70 and ZOTU76. The three investigated *Hylocereus* species also exhibited a unique profile characterized by a combination of ZOTU1, ZOTU2, and ZOTU7 (a derivative of ZOTU2) (Figure 3, Table 1). Among the studied taxa, species of the genus *Deamia* were markedly distinct from *Selenicereus*, *Hylocereus*, and *Epiphyllum* based on their ribotype profiles, characterized by the major ribotype ZOTU3 and its derivative ZOTU6. Furthermore, the distinct pseudogenic variant ZOTU9 was detected in three out of the four analyzed *Deamia* samples.

Our dataset included three *Selenicereus* samples (S31, S65, and S66) whose morphological characteristics suggested a hybrid origin. Two of these samples, S65 and S66, exhibited ribotype profiles indistinguishable from those observed in most other *Selenicereus* samples. Conversely, sample S31 harbored ZOTU1 and ZOTU2 in roughly equal frequencies, indicating that *Selenicereus hamatus* may have been one of the parental species. We did not detect elevated ITS variability or increased ribotype diversity in these putative hybrids.

## 3. Discussion

In an investigation of intragenomic ITS2 polymorphism across 178 plant species, Song et al. [43] reported that these genomes harbored between 1 and 253 distinct ITS2 variants, with a mean of 35 and a median of 23 variants per genome. Similarly, our previous study on intragenomic ITS1 polymorphism in 22 grass species revealed that the number of ribotypes ranged from 1 to 23, with an average of 6.6 variants per genome [44]. Furthermore, 1 to 5 major ribotypes (exceeding 5% of total reads) alongside 3 to 23 minor ribotypes per genome were documented in 15 *Sparganium* L. species (Typhaceae Juss.) [13], whereas 8–11 ribotypes represented by more than 100 reads were detected in the genomes of *Pulsatilla vernalis* (L.) Mill. (Ranunculaceae Juss.) [45]. Against this background, the intragenomic diversity of ribotypes in the examined cacti is remarkably low. Specifically, 44 studied plants contained a single major ribotype, while only two major ribotypes were detected in 9 genomes. Moreover, no minor ribotypes (defined by a minimum read depth of 10) were detected in 24 cacti genomes, 20 genomes harbored only a single minor ribotype, 5 genomes contained two, and 2 genomes possessed three minor variants. Four minor ribotypes were uniquely observed in *Hylocereus trigonus* (Haw.) Saff. S36. These results suggest that the investigated cacti possess an unusually low level of intragenomic rDNA heterogeneity. This pattern is likely attributable to infrequent interspecific hybridization and efficient concerted evolution within rDNA arrays.

Among the samples examined in this study, there were three, or possibly four, putative hybrids (S31, S65, S66, and probably S56). It is well established that hybridization between divergent lineages or distinct species can trigger genome instability [46,47,48,49]. In cacti, such instability can drive the emergence of highly polyploid karyotypes [50]. In our study, however, we did not observe any significant burst of sequence variability within the ITS1 region of these putatively hybrid individuals, nor did their ribotype patterns exhibit elevated diversity.

Cytological observations indicate that several taxa included in this study—namely *Deamia testudo* (Karw. ex Zucc.) Britton & Rose, *Hylocereus guatemalensis* (Eichlam ex Weing.) Britton & Rose, *H. trigonus* (Haw.) Saff., *H. undatus* (Haw.) Britton & Rose, *Selenicereus grandiflorus* (L.) Britton & Rose, *S. grandiflorus* subsp. *hondurensis* (K.Schum. ex Weing.) Ralf Bauer, *S.* aff. *megalanthus* (K.Schum. ex Vaupel) Moran, *S. pteranthus* (Link ex A. Dietr.) Britton & Rose, *S.* aff. *setaceus*, and *S. spinulosus* (DC.) Britton & Rose—are diploid. In contrast, *S. setaceus* and *S. megalanthus* (syn. *H. megalanthus* (K. Schum. ex Vaupel) Ralf Bauer) are tetraploid [50,51,52,53,54,55]. Whole-genome sequencing of the tetraploid *S. megalanthus* suggested that it is an autopolyploid, potentially a segmental allopolyploid or a descendant of a cross between two diverged populations of the same species characterized by genetically distinct but structurally similar chromosomes—an “interracial autopolyploid” [56]. Although there are two NORs per haploid genome in tetraploid *Selenicereus* and *Hylocereus* compared with a single NOR in diploid genomes [54,55], we detected no substantial differences in intragenomic rDNA polymorphism between diploids and polyploids (Table 1).

All minor ribotypes identified in the studied cacti were considered putative pseudogenes, with the majority harboring distinct insertions and deletions. These findings are broadly consistent with those of Plume et al. [37] regarding nrDNA ITS polymorphism in *Hylocereus monacanthus* (hort. ex Lem.) Britton & Rose, *H. megalanthus*, and *Selenicereus hamatus*. These authors demonstrated that the nrDNA ITS amplicons from these three species, sequenced using the standard universal primers ITS4 [57] and ITS5a [58], exhibited substantial length polymorphism.

In the present study, a single primer pair—ITS1P [59] and ITS2 [57]—was utilized for ITS sequence amplification. In our companion study investigating the interspecific polymorphism of the ITS1–5.8S rDNA–ITS2 region in *Selenicereus* [60], we employed a different primer combination: ITS1P and ITS4. Notably, in both studies, we failed to detect the nrDNA ITS pseudogene variants described by Plume et al. [37]. This discrepancy is likely attributable to substantial sequence divergence within the primer-binding sites of these two distinct pseudogene lineages, a hypothesis corroborated by our bioinformatic analysis [60]. When examining intragenomic and interspecific ITS1 variations in *Selenicereus* and *Hylocereus*, we did not encounter the ITS1 variants previously sequenced by Plume et al. [37] among either major or minor ribotypes. We have previously shown that a characteristic 4-nucleotide deletion occurs within the ITSC1-R primer-binding region of these pseudogenes; this deletion is entirely absent from the canonical rDNA sequence on chromosome 11 of *Selenicereus monacanthus* (CM104744.1). Consequently, the ITSC1-F/ITSC1-R primer pair is unlikely to amplify the functional ITS1–5.8S–ITS2 copy from the *Selenicereus monacanthus* NOR [60].

The mutational processes driving the pseudogenization of 35S rRNA genes in cacti encompass the accumulation of single-nucleotide mutations and deletions within both the coding regions and spacers, alongside extensive insertions within the spacers [28,29,32,33,34,37,38]. Consistent with previous reports, sequence comparisons between functional genes and pseudogenes revealed a pronounced transition bias in the pseudogenic copies [39,61]. Furthermore, in line with patterns documented in other plant taxa [28,29,30], a clear trend toward A/T enrichment was observed in pseudogenic ITS1 sequences. It is likely that most, if not all, pseudogenes residing within the NORs are hypermethylated and transcriptionally silent [62]. Cytosine methylation in transcriptionally inactive 35S rDNA pseudogenes may therefore contribute to the progressive loss of cytosine residues in ITS sequences. Specifically, it is well established that cytosine methylation followed by spontaneous deamination results in a C-to-T transition (or a G-to-A transition on the complementary strand) during DNA replication [63,64].

The majority of the minor ribotypes—which putatively represent pseudogenes—harbored deletions spanning the A2, A3, and B1 processing sites. These sites play critical roles in the precise cleavage and maturation of pre-rRNA [3,40]. The recurrent disruption of these processing sites may reflect purifying selection acting to prevent the production of aberrant ribosomes. Pre-rRNA processing constitutes an important checkpoint in ribosome quality control [65]. Indeed, even a small fraction of mutant 18S and 5.8S rRNA molecules may exert partially dominant deleterious effects [26].

## 4. Materials and Methods

### 4.1. Plant Material and Sampling

The intragenomic polymorphism of the 18S rDNA (partial)–ITS1–5.8S rDNA (partial) region was investigated across 25 cactus species and subspecies. Plant tissues were sampled from the living greenhouse collections of the Peter the Great Botanical Garden of the Komarov Botanical Institute of the Russian Academy of Sciences, St. Petersburg (hereafter BIN); the N.V. Tsitsin Main Botanical Garden of the Russian Academy of Sciences, Moscow (hereafter Tsitsin BG); and the Moscow State University Botanical Garden “Apothecary Garden”, Moscow (hereafter MGU).

The examined species, sample codes, and their respective institutional accession numbers are as follows:

- *Deamia chontalensis* (Alexander) Doweld: samples S6 (BIN 10879) and S60 (MGU 17041001);
- *Deamia testudo* (Karw. ex Zucc.) Britton & Rose: samples S1 (BIN 244925), S46 (Tsitsin BG 0000.03910), and S64 (MGU 17041004);
- *Epiphyllum chrysocardium* Alexander: sample S20 (BIN 244951);
- *Hylocereus guatemalensis* (Eichlam ex Weing.) Britton & Rose [syn. of *Selenicereus guatemalensis* (Eichlam ex Weing.) D.R.Hunt]: sample S35 (BIN16031);
- *Hylocereus trigonus* (Haw.) Saff. [syn. of *Selenicereus triangularis* (L.) D.R.Hunt]: samples S33 (Tsitsin BG 0000.03910) and S36 (BIN 17815);
- *Hylocereus undatus* (Haw.) Britton & Rose [syn. of *Selenicereus undatus* (Haw.) D.R.Hunt]: sample S34 (BIN 19042d);
- *Selenicereus anthonyanus* (Alexander) D.R.Hunt: samples S12 (BIN 244969) and S63 (MGU 17129002);
- *Selenicereus dorschianus* Ralf Bauer: sample S57 (BIN 17129007);
- *Selenicereus grandiflorus* (L.) Britton & Rose subsp. *grandiflorus*: samples S7 (BIN 8626), S15 (BIN 8648), S16 (BIN 244884), S32 (BIN 160442d), S43 (Tsitsin BG2017.04293), and S67 (BIN 17129011);
- *Selenicereus grandiflorus* subsp. *donkelaarii* (Salm-Dyck) Ralf Bauer: sample S5 (BIN 8637);
- *Selenicereus grandiflorus* subsp. *hondurensis* (K.Schum. ex Weing.) Ralf Bauer: samples S3 (BIN 7141), S52 (MGU 171290162B), and S53 (MGU 171290162A);
- *Selenicereus hamatus* (Scheidw.) Britton & Rose: samples S9 (BIN 13349) and S41 (Tsitsin BG 0000.03904);
- *Selenicereus inermis* (Otto ex Pfeiff.) Britton & Rose: sample S18 (BIN 263408);
- *Selenicereus murrillii* Britton & Rose: samples S2 (BIN 251668) and S55 (MGU 17129020);
- *Selenicereus nelsonii* (Weing.) Britton & Rose: sample S8 (BIN 241128);
- *Selenicereus pteranthus* (Link ex A.Dietr.) Britton & Rose: samples S17 (BIN 7427), S45 (Tsitsin BG 0000.03908), and S58 (Tsitsin BG 17129022);
- *Selenicereus pteranthus* f. *macdonaldiae* (Hook.) Ralf Bauer: samples S14 (BIN 241876), S44 (Tsitsin BG 0000.03906), and S56 (MGU 171290221);
- *Selenicereus megalanthus* (K.Schum. ex Vaupel) Moran: sample S51 (BIN 17129016);
- *Selenicereus setaceus* (Salm-Dyck ex DC.) Werderm.: samples S13 (BIN 16793), S68 (BIN 17129025a), and S69 (BIN 17129025b);
- *Selenicereus spinulosus* (DC.) Britton & Rose: samples S4 (BIN 12329d) and S54 (MGU 17129026);
- *Selenicereus urbanianus* (Gürke & Weing.) Britton & Rose: sample S11 (BIN 8495);
- *Selenicereus vagans* (K.Brandegee) Britton & Rose: samples S10 (BIN 94233), S40 (Tsitsin BG 0000.03911), and S62 (MGU 17129033);
- *Selenicereus validus* S.Arias & U.Guzmán: samples S19 (BIN 8657) and S59 (BIN 17129034);
- *Selenicereus wercklei* (F.A.C.Weber) Britton & Rose: samples S50 (MGU 17129015B) and S61 (BIN 17129015A);
- *Selenicereus* sp.: samples S31 (*S. grandiflorus × S. hamatus*?) (BIN 3516), S65 (MGU 17129000A), and S66 (MGU 17129021x).

### 4.2. DNA Extraction, PCR Amplification, and Next-Generation Sequencing

Genomic DNA was isolated from fresh plant material using the Qiagen DNeasy Plant Mini Kit (Qiagen Inc., Hilden, Germany) following the manufacturer’s protocol. For the amplification of the 18S rDNA (fragment)–ITS1–5.8S rDNA (fragment) region, the PCR amplification was performed under following conditions: initial denaturation at 94 °C for 1 min; followed by 35 cycles of denaturation at 94 °C for 30 s, annealing at 55 °C for 30 s, and extension at 72 °C for 30 s; with a final elongation step at 72 °C for 5 min. Amplification was performed using the ITS1P [59] and ITS2 [57] primers. PCR amplifications were carried out at the Core Facility “Cellular and Molecular Technologies for the Study of Plants and Fungi” of the Komarov Botanical Institute (St. Petersburg, Russia).

The resulting PCR products were purified using Agencourt AMPure XP magnetic beads (Beckman Coulter, Indianapolis, IN, USA). Sequencing libraries were constructed according to the MiSeq Reagent Kit Preparation Guide (Illumina). High-throughput sequencing was performed on an Illumina MiSeq instrument (Illumina, San Diego, CA, USA) using the MiSeq Reagent Kit v3 (600 cycles) with paired-end reads (2 × 300 bp) according to the manufacturer’s instructions. Library preparation and sequencing were performed at the Core Facility “Genomic Technologies, Proteomics, and Cell Biology” of the All-Russian Research Institute of Agricultural Microbiology (St. Petersburg, Russia). The NGS sequence data for the ZOTUs have been deposited in NCBI GenBank under accession numbers: ZOTU1 PZ686292, ZOTU2 PZ686293, ZOTU3 PZ686294, ZOTU4 PZ686295, ZOTU6 PZ686296, ZOTU7 PZ686297, ZOTU8 PZ686298, ZOTU9 PZ686299, ZOTU23 PZ686300, ZOTU57 PZ686301, ZOTU70 PZ686302, ZOTU75 PZ686303, ZOTU76 PZ686304, ZOTU81 PZ686305 ZOTU96 PZ686306, ZOTU107 PZ686307, ZOTU108 PZ686308, ZOTU109 PZ686309, and ZOTU112 PZ686310.

### 4.3. Molecular Phylogenetic Analysis

Raw paired-end reads were processed and quality-trimmed using a sliding-window approach rimmed using Trimmomatic [66] integrated within the Unipro UGENE Raw paired-end reads were processed and quality-trimmed using a sliding-window approach rimmed using Trimmomatic [66] integrated within the Unipro UGENE (Novosibirsk Center of Information Technologies LLC, Novosibirsk, Russia) toolkit) toolkit [67]. Trimming employed a sliding window approach (window size 4, quality threshold 12) and a minimum read length cutoff of 130 bp. Paired reads were subsequently merged; dereplication and abundance-based sorting were performed using VSEARCH v2.7.1 (Department of Informatics, University of Oslo, Oslo, Norway) [68]. The recovered sequences were treated as zero-radius operational taxonomic units (ZOTUs or ribotypes) representing the genomic rDNA pool and were ranked by frequency. To ensure sequence reliability and exclude potential artifacts, we applied a minimum abundance threshold of 10 reads per ZOTU within each sample.

Multiple sequence alignments were constructed using MEGA 12 (Temple University, Philadelphia, PA, USA) [69]. A ribotype network was reconstructed using statistical parsimony in TCS v1.2.1 (Brigham Young University, Provo, UT, USA) [41] and subsequently visualized using tcsBU (University of Porto, Porto, Portugal) [42]. Additionally, phylogenetic relationships among the obtained ribotypes were inferred using both Maximum Likelihood (ML) under the TIM+I substitution model (Tamura three-parameter model with a proportion of invariant sites) and Bayesian inference (BI). ML analyses were conducted in IQ-TREE v2.3.6. (Computational Molecular Evolution Group, Center for Integrative Bioinformatics Vienna (CIBIV), University of Vienna, Vienna, Austria) [70], BI was performed in MrBayes v3.2.2 [71] for 2–5 million generations; the evolutionary distances were calculated using the two-parameter Kimura model (K2P) (MEGA 12) [72]. ML and Bayesian inference gave the same topology. The pairwise deletion option was used to handle ambiguous positions and gaps for each sequence pair, resulting in a final aligned dataset comprising 430 positions.

## 5. Conclusions

Our investigation of intragenomic rDNA polymorphism in several cactus species of the genera *Selenicereus* (sensu stricto), former *Hylocereus* (now included within *Selenicereus*), *Epiphyllum*, and *Deamia* demonstrates that intragenomic rDNA diversity in these taxa is lower than that previously reported for *Lophocereus* and *Pachycereus* [34]. This pattern is consistent with relatively infrequent interspecific hybridization and efficient concerted evolution within their rDNA arrays.

The observed polymorphism occurred in two principal forms. First, several genomes contained major ribotypes characteristic of two different species in approximately equal proportions, suggesting a hybrid origin. Second, some genomes harbored low-frequency 35S rDNA variants carrying deletions within the coding regions of 18S and 5.8S rDNA together with extensive insertions and deletions in ITS1. Many of these mutations disrupted the conserved A2, A3, and B1 processing sites required for proper pre-rRNA maturation [3,40]. Consequently, the products of these putative pseudogenes could not participate in ribosome formation [65].

## Figures and Tables

**Table 1:** (**A**,**B**). Interspecific and intragenomic polymorphism of ITS1 ribotypes in 25 species of the tribe Phyllocacteae revealed by locus-specific next-generation sequencing (NGS) on the Illumina platform; (**A**)—variant abundance (number of reads) of various ITS1 variants in genome, (**B**)—relative variant abundance (% reads) of various ITS1 variants in genome, where bold signifies a ‘‘major variant’’ that was defined as any variant whose relative abundance is greater than 5%.

(A) Species and Voucher	Variant Abundance (Number of Reads) of Various ITS1 Variants in Genome																		
	ZOTU1	ZOTU2	ZOTU3	ZOTU4	ZOTU6	ZOTU7	ZOTU8	ZOTU9	ZOTU23	ZOTU57	ZOTU70	ZOTU75	ZOTU76	ZOTU81	ZOTU96	ZOTU107	ZOTU108	ZOTU109	ZOTU112
***Deamia chontalensis*** **S6**	0	0	3187	0	0	0	0	170	0	0	0	0	0	0	0	0	0	0	0
***Deamia chontalensis*** **S60**	0	0	3105	0	0	0	0	228	0	0	0	0	0	0	0	0	0	0	0
***Deamia testudo*** **S1**	0	0	0	0	5597	0	0	0	0	0	0	0	0	0	0	0	0	0	0
***Deamia testudo*** **S46**	0	0	4733	0	0	0	0	711	0	0	0	0	0	0	0	0	0	0	0
***Deamia testudo*** **S64**	10	0	4176	0	0	0	0	761	0	0	0	0	0	0	0	0	0	0	0
***Epiphyllum chrysocardium*** **S20**	96	0	0	9487	0	0	0	0	0	0	31	0	25	0	0	0	0	0	0
***Hylocereus guatemalensis*** **S35**	1690	3512	0	0	0	0	0	0	0	0	0	0	0	0	0	0	0	0	0
***Hylocereus trigonus*** **S33**	18	2053	0	0	0	3490	0	0	0	0	0	0	0	0	13	0	0	0	0
***Hylocereus trigonus*** **S36**	55	6812	0	0	0	100	0	0	0	0	0	0	0	10	24	0	0	0	0
***Hylocereus undatus*** **S34**	24	1046	0	0	0	1983	0	0	0	0	0	0	0	0	0	0	0	0	0
***Selenicereus anthonyanus*** **S12**	7701	0	0	0	0	0	0	0	29	0	0	0	0	0	0	0	0	0	0
***Selenicereus anthonyanus*** **S63**	5907	0	0	0	0	0	0	0	13	0	0	0	0	0	31	0	0	0	0
***Selenicereus dorschianus*** **S57**	5981	0	0	0	0	0	0	0	0	0	0	0	0	0	10	0	0	0	0
***Selenicereus grandiflorus*** **S7**	5463	0	0	0	0	0	0	0	0	0	0	0	0	0	0	0	0	0	0
***Selenicereus grandiflorus*** **S16**	7166	0	0	0	0	0	0	0	0	0	0	0	0	0	0	0	0	0	0
***Selenicereus grandiflorus*** **S32**	5222	0	0	0	0	0	0	0	0	0	0	0	0	0	0	0	0	0	0
***Selenicereus grandiflorus*** **S43**	6460	0	24	0	0	0	0	0	0	0	0	0	0	0	0	0	0	0	0
***Selenicereus grandiflorus*** **S67**	5974	0	0	0	0	0	0	0	0	0	0	15	0	0	12	0	0	0	0
***S. grandiflorus*** **ssp. *****grandiflorus***** S15**	6178	0	0	0	0	0	0	0	0	0	0	0	0	0	0	0	0	0	0
***S. grandiflorus*** **ssp. *****donkelaarii***** S5**	2913	0	0	0	0	0	0	0	0	0	0	0	0	0	0	0	0	0	0
***S. grandiflorus*** **ssp. *****hondurensis***** S3**	6569	0	0	0	0	0	0	0	0	0	0	0	0	0	0	0	0	0	0
***S. grandiflorus*** **ssp. *****hondurensis***** S52**	2910	0	0	0	0	0	0	0	0	0	0	0	0	0	0	0	0	0	0
***S. grandiflorus*** **ssp. *****hondurensis***** S53**	6020	0	0	0	0	0	0	0	0	0	0	0	0	0	0	0	0	0	0
***Selenicereus hamatus*** **S9**	36	8702	0	0	0	0	0	0	0	0	0	0	0	0	0	0	0	0	0
***Selenicereus hamatus*** **S41**	59	8190	0	0	0	0	0	0	0	0	0	0	0	14	0	0	0	0	0
***Selenicereus inermis*** **S18**	9094	0	0	0	0	0	12	0	0	0	0	0	0	0	0	0	0	0	0
***Selenicereus murrillii*** **S2**	6373	0	0	0	0	0	0	0	0	0	0	0	0	0	0	0	0	0	0
***Selenicereus murrillii*** **S55**	7232	11	0	0	0	0	0	0	0	0	0	0	0	0	0	0	0	0	0
***Selenicereus nelsonii*** **S8**	6989	0	0	0	0	0	0	0	0	0	0	0	0	0	14	0	0	0	0
***Selenicereus pteranthus*** **S17**	8656	0	0	0	0	0	0	0	0	12	0	0	0	0	0	0	0	0	0
***Selenicereus pteranthus*** **S45**	5378	0	0	0	0	0	0	0	0	0	0	0	0	0	0	0	0	0	0
***Selenicereus pteranthus*** **S58**	5468	0	0	0	0	0	0	0	0	0	0	0	0	0	0	0	0	0	0
***S. pteranthus*** **f. *****macdonaldiae***** S14**	5600	0	0	0	0	0	0	0	0	0	0	0	0	0	0	0	0	0	0
***S. pteranthus*** **f. *****macdonaldiae***** S56**	2598	3376	0	0	0	0	0	0	0	0	0	0	0	0	0	0	0	0	0
***S. pteranthus*** **f. *****macdonaldiae***** S44**	5793	0	0	0	0	0	0	0	0	0	0	0	0	0	0	0	12	0	0
***Selenicereus megalanthus*** **S51**	5589	0	0	18	0	0	0	0	0	0	0	0	0	0	14	0	0	0	0
***Selenicereus setaceus*** **S13**	7413	0	0	0	0	0	0	0	0	0	0	0	0	0	0	0	0	0	0
***Selenicereus setaceus*** **S68**	77	0	0	0	0	0	5649	0	0	0	0	0	0	0	0	0	0	0	0
***Selenicereus setaceus*** **S69**	5389	0	0	0	0	0	0	0	0	0	0	0	0	0	27	0	0	0	0
***Selenicereus spinulosus*** **S4**	5169	0	0	0	0	0	0	0	0	0	0	0	0	0	0	0	0	0	0
***Selenicereus spinulosus*** **S54**	5063	0	0	0	0	0	0	0	0	0	0	0	0	0	13	0	0	0	0
***Selenicereus spinulosus*** **S42**	7098	0	0	0	0	0	0	0	0	0	0	0	0	0	0	0	0	0	0
***Selenicereus urbanianus*** **S11**	9073	0	0	0	0	0	0	0	0	13	0	0	0	0	0	0	0	0	0
***Selenicereus vagans*** **S10**	8684	0	0	0	0	0	0	0	0	0	0	0	0	0	0	0	0	0	11
***Selenicereus vagans*** **S40**	7749	0	0	0	0	0	0	0	0	0	0	0	0	0	0	0	0	0	0
***Selenicereus vagans*** **S62**	6155	0	0	0	0	0	0	0	0	0	0	0	0	0	0	13	0	12	0
***Selenicereus validus*** **S19**	7857	0	0	0	0	0	0	0	0	0	0	0	0	0	0	0	0	0	0
***Selenicereus validus*** **S59**	6399	0	0	0	0	0	0	0	0	0	0	0	0	0	10	0	0	0	0
***Selenicereus wercklei*** **S50**	6726	0	0	0	0	0	0	0	0	10	0	0	0	0	0	0	0	0	0
***Selenicereus wercklei*** **S61**	6422	0	0	0	0	0	17	0	343	0	0	0	0	0	0	0	0	0	0
***Selenicereus*** **sp. S65**	7189	0	0	0	0	0	0	0	0	12	0	12	0	0	15	0	0	0	0
***Selenicereus*** **sp. S66**	5632	0	0	0	0	0	0	0	0	0	0	0	0	0	14	0	0	0	0
***Selenicereus*** **sp. S31**	1589	1850	0	0	0	0	0	0	0	0	0	0	0	0	0	0	0	0	0
**(B)** **Species and Voucher**	**Relative Variant Abundance (% Reads) of Various ITS1 Variants in Genome**																		
	**ZOTU1**	**ZOTU2**	**ZOTU3**	**ZOTU4**	**ZOTU6**	**ZOTU7**	**ZOTU8**	**ZOTU9**	**ZOTU23**	**ZOTU57**	**ZOTU70**	**ZOTU75**	**ZOTU76**	**ZOTU81**	**ZOTU96**	**ZOTU107**	**ZOTU108**	**ZOTU109**	**ZOTU112**
***Deamia chontalensis*** **S6**	0	0	**94.9**	0	0	0	0	**5.1**	0	0	0	0	0	0	0	0	0	0	0
***Deamia chontalensis*** **S60**	0	0	**93.2**	0	0	0	0	**6.8**	0	0	0	0	0	0	0	0	0	0	0
***Deamia testudo*** **S1**	0	0	0	0	**100**	0	0	0	0	0	0	0	0	0	0	0	0	0	0
***Deamia testudo*** **S46**	0	0	**86.9**	0	0	0	0	**13.1**	0	0	0	0	0	0	0	0	0	0	0
***Deamia testudo*** **S64**	0.2	0	**84.4**	0	0	0	0	**15.4**	0	0	0	0	0	0	0	0	0	0	0
***Epiphyllum chrysocardium*** **S20**	1	0	0	**98.4**	0	0	0	0	0	0	0.3	0	0.3	0	0	0	0	0	0
***Hylocereus guatemalensis*** **S35**	**32.5**	**67.5**	0	0	0	0	0	0	0	0	0	0	0	0	0	0	0	0	0
***Hylocereus trigonus*** **S33**	0.3	**36.8**	0	0	0	**62.6**	0	0	0	0	0	0	0	0	0.3	0	0	0	0
***Hylocereus trigonus*** **S36**	0.8	**97.3**	0	0	0	1.4	0	0	0	0	0	0	0	0.1	0.4	0	0	0	0
***Hylocereus undatus*** **S34**	0.8	**34.2**	0	0	0	**65**	0	0	0	0	0	0	0	0	0	0	0	0	0
***Selenicereus anthonyanus*** **S12**	**99.6**	0	0	0	0	0	0	0	0.4	0	0	0	0	0	0	0	0	0	0
***Selenicereus anthonyanus*** **S63**	**99.3**	0	0	0	0	0	0	0	0.2	0	0	0	0	0	0.5	0	0	0	0
***Selenicereus dorschianus*** **S57**	**99.8**	0	0	0	0	0	0	0	0	0	0	0	0	0	0.2	0	0	0	0
***Selenicereus grandiflorus*** **S7**	**100**	0	0	0	0	0	0	0	0	0	0	0	0	0	0	0	0	0	0
***Selenicereus grandiflorus*** **S16**	**100**	0	0	0	0	0	0	0	0	0	0	0	0	0	0	0	0	0	0
***Selenicereus grandiflorus*** **S32**	**100**	0	0	0	0	0	0	0	0	0	0	0	0	0	0	0	0	0	0
***Selenicereus grandiflorus*** **S43**	**99.6**	0	0.4	0	0	0	0	0	0	0	0	0	0	0	0	0	0	0	0
***Selenicereus grandiflorus*** **S67**	**99.6**	0	0	0	0	0	0	0	0	0	0	0.2	0	0	0.2	0	0	0	0
***S. grandiflorus*** **ssp. *****grandiflorus***** S15**	**100**	0	0	0	0	0	0	0	0	0	0	0	0	0	0	0	0	0	0
***S. grandiflorus*** **ssp. *****donkelaarii***** S5**	**100**	0	0	0	0	0	0	0	0	0	0	0	0	0	0	0	0	0	0
***S. grandiflorus*** **ssp. *****hondurensis***** S3**	**100**	0	0	0	0	0	0	0	0	0	0	0	0	0	0	0	0	0	0
***S. grandiflorus*** **ssp. *****hondurensis***** S52**	**100**	0	0	0	0	0	0	0	0	0	0	0	0	0	0	0	0	0	0
***S. grandiflorus*** **ssp. *****hondurensis***** S53**	**100**	0	0	0	0	0	0	0	0	0	0	0	0	0	0	0	0	0	0
***Selenicereus hamatus*** **S9**	0.4	**99.6**	0	0	0	0	0	0	0	0	0	0	0	0	0	0	0	0	0
***Selenicereus hamatus*** **S41**	0.7	**99.1**	0	0	0	0	0	0	0	0	0	0	0	0.2	0	0	0	0	0
***Selenicereus inermis*** **S18**	**99.9**	0	0	0	0	0	0.1	0	0	0	0	0	0	0	0	0	0	0	0
***Selenicereus murrillii*** **S2**	**100**	0	0	0	0	0	0	0	0	0	0	0	0	0	0	0	0	0	0
***Selenicereus murrillii*** **S55**	**99.9**	0.1	0	0	0	0	0	0	0	0	0	0	0	0	0	0	0	0	0
***Selenicereus nelsonii*** **S8**	**99.8**	0	0	0	0	0	0	0	0	0	0	0	0	0	0.2	0	0	0	0
***Selenicereus pteranthus*** **S17**	**99.9**	0	0	0	0	0	0	0	0	0.1	0	0	0	0	0	0	0	0	0
***Selenicereus pteranthus*** **S45**	**100**	0	0	0	0	0	0	0	0	0	0	0	0	0	0	0	0	0	0
***Selenicereus pteranthus*** **S58**	**100**	0	0	0	0	0	0	0	0	0	0	0	0	0	0	0	0	0	0
***S. pteranthus*** **f. *****macdonaldiae***** S14**	**100**	0	0	0	0	0	0	0	0	0	0	0	0	0	0	0	0	0	0
***S. pteranthus*** **f. *****macdonaldiae***** S56**	**43.5**	**56.5**	0	0	0	0	0	0	0	0	0	0	0	0	0	0	0	0	0
***S. pteranthus*** **f. *****macdonaldiae***** S44**	**99.8**	0	0	0	0	0	0	0	0	0	0	0	0	0	0	0	0.2	0	0
***Selenicereus megalanthus*** **S51**	**99.4**	0	0	0.3	0	0	0	0	0	0	0	0	0	0	0.3	0	0	0	0
***Selenicereus setaceus*** **S13**	**100**	0	0	0	0	0	0	0	0	0	0	0	0	0	0	0	0	0	0
***Selenicereus setaceus*** **S68**	1.3	0	0	0	0	0	**98.7**	0	0	0	0	0	0	0	0	0	0	0	0
***Selenicereus setaceus*** **S69**	**99.5**	0	0	0	0	0	0	0	0	0	0	0	0	0	0.5	0	0	0	0
***Selenicereus spinulosus*** **S4**	**100**	0	0	0	0	0	0	0	0	0	0	0	0	0	0	0	0	0	0
***Selenicereus spinulosus*** **S54**	**99.8**	0	0	0	0	0	0	0	0	0	0	0	0	0	0.2	0	0	0	0
***Selenicereus spinulosus*** **S42**	**100**	0	0	0	0	0	0	0	0	0	0	0	0	0	0	0	0	0	0
***Selenicereus urbanianus*** **S11**	**99.9**	0	0	0	0	0	0	0	0	0.1	0	0	0	0	0	0	0	0	0
***Selenicereus vagans*** **S10**	**99.9**	0	0	0	0	0	0	0	0	0	0	0	0	0	0	0	0	0	0.1
***Selenicereus vagans*** **S40**	**100**	0	0	0	0	0	0	0	0	0	0	0	0	0	0	0	0	0	0
***Selenicereus vagans*** **S62**	**99.6**	0	0	0	0	0	0	0	0	0	0	0	0	0	0	0.2	0	0.2	0
***Selenicereus validus*** **S19**	**100**	0	0	0	0	0	0	0	0	0	0	0	0	0	0	0	0	0	0
***Selenicereus validus*** **S59**	**99.8**	0	0	0	0	0	0	0	0	0	0	0	0	0	0.2	0	0	0	0
***Selenicereus wercklei*** **S50**	**99.9**	0	0	0	0	0	0	0	0	0.1	0	0	0	0	0	0	0	0	0
***Selenicereus wercklei*** **S61**	**94.7**	0	0	0	0	0	0.3	0	**5**	0	0	0	0	0	0	0	0	0	0
***Selenicereus*** **sp. S65**	**99.4**	0	0	0	0	0	0	0	0	0.2	0	0.2	0	0	0.2	0	0	0	0
***Selenicereus*** **sp. S66**	**99.8**	0	0	0	0	0	0	0	0	0	0	0	0	0	0.2	0	0	0	0
***Selenicereus*** **sp. S31**	**46.2**	**53.8**	0	0	0	0	0	0	0	0	0	0	0	0	0	0	0	0	0

**Table 2:** Ribotype polymorphism in cacti: transitions, transversions, deletions, and insertions relative to ZOTU1 (63,3%GC).

Zotu	%GC	SNPs (P/Q)				Deletion (bp)				Insertion (bb)
		18S	ITS1	5.8S	Sum	18S	ITS1	5.8S	Sum	ITS1
Zotu2	63.6	-	1/0	-	1/0	-	-	-	-	-
Zotu3	63.4	-	4/2	-	4/2	-	1	-	1	1
Zotu4	63.3	-	2/1	-	2/1	-	-	-	-	-
Zotu6	62.8	-	5/3	-	5/3	-	1	-	1	1 + 1
Zotu7	63.0	-	3/0	-	3/0	-	-	-	--	-
Zotu8	63.0	-	1/1	-	1/1	-	-	-	-	-
					**Putative pseudogenes**					
Zotu9	56.4	1/3	6/9	-	7/12	4	29 + 21 + 1 + 62 + 5	5 + 5	78	46 + 50
Zotu81	58.2	0/1	1/0	0/9	1/10	-	-	-	-	-
Zotu57	63.3	0/1	0/1	0/8	0/10	-	-	-	-	-
Zotu75	52.5	-	3/0	2/0	5/0	-	63	11	74	-
Zotu107	57.9	6/0	26/3	1/0	33/3	-	1	-	-	1
Zotu70	61.9	2/0	23/3	-	25/3	-	1 + 1+41	30 + 1	74	1
Zotu109	63.6	4/0	19/1	1/1	24/2	-	-	-	-	-
Zotu96	63.1	1/3	0/2	-	1/5	4	-	-	-	-
Zotu23	55.7	-	0/1	-	0/1	-	45	-	45	-
Zotu76	59.1	-	2/1	-	2/1	-	46	-	46	-
Zotu108	57.5	-	0/1	-	0/1	-	15 + 21	-	36	-
Zotu112	58.1	-	1/0	-	1/0	-	92	-	92	-
Sum		13/5	97/29	4/18	114/52					

## Data Availability

All data generated or analyzed during this study are contained within the article.
